# The H2TH-like motif of the *Escherichia coli* multifunctional protein KsgA is required for DNA binding involved in DNA repair and the suppression of mutation frequencies

**DOI:** 10.1186/s41021-023-00266-5

**Published:** 2023-04-12

**Authors:** Yuichiro Hayashi, Masafumi Funakoshi, Kaname Hirosawa, Qiu-Mei Zhang-Akiyama

**Affiliations:** 1 Laboratory of Stress Response Biology, Graduate School of Science, Kyoto University, Kitashirakawa Oiwake-cho, Sakyo-ku, Kyoto, 606-8502 Japan; 2 Takara Bio Inc., Nojihigashi, Kusatsu-shi, Shiga 525-0058 Japan; 3 Department of Biosystems Science, Institute for Frontier Life and Medical Sciences, Kyoto University, Sakyo, Kyoto, 606-8507 Japan

**Keywords:** KsgA, DNA binding, Methyltransferase, H2TH domain, 3D structure

## Abstract

**Background:**

DNA oxidatively damaged by reactive oxygen species is repaired by base excision repair (BER) pathway proteins, with DNA glycosylases removing damaged or mismatched bases in the first step of BER. KsgA is a multifunctional protein that exhibits the activities of two enzymes, DNA glycosylase and rRNA dimethyltransferase. The structure-function relationship of the KsgA protein in cellular DNA repair remains unclear because the domains required for KsgA to recognize DNA have not been identified.

**Purpose:**

To clarify the mechanisms by which KsgA recognizes damaged DNA and to identify the DNA-binding site, which exists in KsgA.

**Methods:**

A structural analysis and in vitro DNA-protein binding assay were performed. The C-terminal function of the KsgA protein was investigated in vitro and in vivo*.*

**Results:**

The 3D conformations of KsgA, MutM, and Nei were compared at UCSF Chimera. The root mean square deviation of KsgA (214-273) and MutM (148-212) and that of KsgA (214-273) and Nei (145-212) were 1.067 and 1.188 Å, both less than 2 Å, suggesting that the C terminal of KsgA is spatially similar to the H2TH domains of MutM and Nei. The full-length KsgA protein and KsgA lacking 1-8 or 214-273 amino acids were purified and used in gel mobility shift assays. KsgA exhibited DNA-binding activity, which was lost in the C-terminally deleted KsgA protein. Spontaneous mutation frequency was measured using a *mutM mutY ksgA*-deficient strain, and the results obtained showed that the mutation frequency was not suppressed by KsgA lacking the C-terminal region, whereas it was in KsgA. To assess dimethyltransferase activity, kasugamycin sensitivity was assessed in wild-type and *ksgA*-deficient strains. Plasmids carrying the full-length *ksgA* gene and C-terminal deletion gene were introduced into *ksgA*-deficient strains. KsgA lacking the C terminus restored dimethyltransferase activity in the *ksgA*-deficient strain as well as KsgA.

**Conclusion:**

The present results confirmed that one enzyme exhibited two activities and revealed that the C-terminal (214-273) amino acids of KsgA were highly similar to the H2TH structural domain, exhibited DNA-binding activity, and inhibited spontaneous mutations. This site is not essential for dimethyltransferase activity.

## Introduction

Reactive oxygen species (ROS) are continuously produced in cells by external factors (including ionizing radiation and chemicals) and internal factors (such as intracellular metabolism). The excessive accumulation of ROS in cells leads to various types of oxidative DNA damage [1–3], which causes mutations, cell death, cancer, and aging [4–6]. For example, 5-formyluracil (5-foU), oxidized thymine, is a representative damaged base. 5-foU in DNA causes mutations as it pairs with not only adenine, but also cytosine and guanine [7, 8]. Thymine glycol (Tg), another type of oxidatively damaged thymine, blocks DNA replication due to its non-planar structure [9, 10]. 5-Hydroxycytosine (5-ohC), oxidized cytosine, causes a mutation by pairing with adenine and guanine [11, 12].

Base excision repair (BER) is an important mechanism that removes damaged bases from DNA [13]. DNA glycosylase, an enzyme that functions in the first step of the BER pathway, removes damaged bases from DNA, generating an apurinic/apyrimidinic (AP) site, and this is followed by repair by AP endonuclease, DNA polymerase, and DNA ligase. DNA glycosylases may be categorized into monofunctional enzymes or bifunctional enzymes based on their activity. Monofunctional DNA glycosylases only exhibit DNA glycosylase activity, which removes bases from DNA backbone to generate AP sites. Bifunctional DNA glycosylases also exhibit AP lyase activity, which cleaves the DNA strand of AP sites. DNA glycosylases have been classified into the following four superfamilies based on their structures and whether they are monofunctional or bifunctional: the uracil DNA glycosylase superfamily, Helix-hairpin-Helix superfamily, methylpurine-DNA glycosylase superfamily, and Fpg/Nei superfamily [14].

The Fpg/Nei superfamily is characterized by its enzymes having a helix-2turn-helix (H2TH) domain. The H2TH domain is necessary for enzymes in this superfamily to bind to DNA and consists of two turns between the two helices [15]. Additionally, most of the enzymes belonging to this superfamily share the common structure of a zinc finger, which is required for DNA binding, and the N-terminal proline or valine as a nucleophile in the DNA glycosylase/AP lyase reaction [16–18]. *Escherichia coli* (*E. coli*) MutM (Fpg), Nei, human NEIL1, NEIL2, and NEIL3 also belong to this superfamily and are all bifunctional enzymes. MutM removes 8-oxo-7,8-dihydroguanine, 4,6-diamino-5-formamidopyrimidine, 2,6-diamino-4-hydroxy-5-formamidopyrimidine, 5-foU, and 5-ohC, while Nei and its homologs remove oxidized pyrimidines [19–26].

*E. coli* KsgA has been identified as a bifunctional DNA glycosylase that removes cytosine paired with oxidized thymine [27]. Previous studies demonstrated that KsgA was associated with oxidative stress. In *Staphylococcus aureus* and *Salmonella enteritidis*, *ksgA* deficiency reduced resistance to oxidative stress [28, 29]. KsgA has been shown to possess a sequence in its C terminus that is similar to the H2TH domain, which is a characteristic of enzymes belonging to the Fpg/Nei superfamily [27]. KsgA is also a ribosomal protein with rRNA dimethyltransferase activity, a deficiency in which increases resistance to kasugamycin [30, 31]. KsgA regulates ribosomal biogenesis by the methylation of the highly conserved 1518th and 1519th adenines at the 3′-end of 16S rRNA [32–34]. This methyltransferase is widely conserved from bacteria to eukaryotes [35–39]. Similar to KsgA, other ribosomal proteins exhibit activities related to BER. The ribosomal protein *Drosophila melanogaster* P0 has been shown to exhibit AP endonuclease activity [40], as has rat rpS3 expressed *in E. coli* [41].

The involvement of ribosomal proteins in BER has not yet been elucidated in detail. The identification of sites that only function in DNA repair will contribute to a more detailed understanding of the mechanisms by which ribosomal proteins function in BER. Therefore, further studies are warranted to identify which amino acid sequences in KsgA are required for the recognition and repair of DNA.

In the present study, we investigated whether the C-terminal region of KsgA with a similar sequence to the H2TH domain contributes to DNA repair. We initially analyzed the 3D structure of its C terminus and revealed that it was structurally similar to the H2TH domains of MutM and Nei. We then confirmed that C-terminal-deficient KsgA did not bind to DNA using a gel mobility shift assay. Furthermore, based on mutation frequencies using rifampicin, C-terminal-deficient KsgA did not suppress spontaneous mutations in *E. coli mutM mutY ksgA*. The kasugamycin assay also revealed that C-terminal-deficient KsgA retained its dimethyltransferase activity. The present study is the first to identify the DNA recognition domain from the multifunctional ribosome protein, demonstrating that one enzyme exhibits two activities.

## Materials and methods

### Protein structure visualization and analysis

Protein structure data were obtained from PDBj (https://pdbj.org/) and analyzed using University of California, San Francisco (UCSF) Chimera (available at http://www.cgl.ucsf.edu/chimera). The data IDs of each protein are as follows: KsgA (PDBID: 1QYR), Nei (PDBID: 1Q39), and MutM (PDBID: 1R2Z).

### Cloning of KsgA and recombinant proteins

To prepare an expression plasmid vector for His-KsgA, His-KsgA (1-213), and His-KsgA (9-273), KsgA, KsgA (1-213), and KsgA (9-273) cDNA fragments were PCR amplified from the pGEX-4 T-3-KsgA plasmid vector using the PCR primers 5′- ATATGGATCCATGAATAATCGAGTCCACCAGG − 3′ (forward) and 5′- GATGCTCGAGACTCTCCTGCAAAGGCG − 3′ (reverse), 5′- ATATGGATCCATGAATAATCGAGTCCACCAGG − 3′ (forward) and 5′- GGTGCTCGAGGATGCGGCTCAACA − 3′ (reverse), and 5′- ATATGGATCCATGCACTTAGCCCGTAAACG − 3′ (forward) and 5′- GATGCTCGAGACTCTCCTGCAAAGGCG − 3′ (reverse), respectively. The amplified fragment containing BamHI and XhoI sites at both ends was inserted into a pET21a (+) vector (National Institute of Genetics). In addition, to prepare an expression plasmid vector for KsgA and KsgA (1-213), KsgA and KsgA (1-213) cDNA fragments were PCR amplified from the pGEX-4 T-3-KsgA plasmid vector using the PCR primers 5′- CGGAATTCATGAATAATCGAGTCCA − 3′ (forward) and 5′-CCCAAGCTTTTAACTCTCCTGCAAAG-3′ (reverse), and 5′- CGGAATTCATGAATAATCGAGTCCA − 3′ (forward) and 5′- CCCAAGCTTTTAGATGCGGCTCAACA − 3′ (reverse), respectively. The amplified fragment containing the EcoRI and HindIII sites at both ends, respectively, was inserted into a pYP73 plasmid vector [42].

### Expression and purification of *E. coli* KsgA

*E. coli* BL21(DE3) (fhuA2 [lon] ompT gal (λ DE3) [dcm] ∆hsdS λ DE3 = λ sBamHIo ∆EcoRI-B int::(lacI::PlacUV5::T7 gene1) i21 ∆nin5) was transformed with the plasmid pET21a(+) containing genes coding KsgA, KsgA (1-213), or KsgA (9-273). Cells were grown at 37 °C in Luria-Bertani (LB) medium containing ampicillin (100 μg/ml, Nacalai Tesque) until optical density at 600 nm (OD_600_) reached approximately 0.5. After the addition of 0.1 mM isopropyl-D-1-thiogalactopyranoside (IPTG), the culture was further incubated at 20 °C for 18 h. Cells were harvested and resuspended in extraction buffer [10 mM Tris-HCl (pH 7.5) containing 5% glycerol, 100 mM NaCl, 10 mM imidazole, 1 mM dithiothreitol (DTT), and 1 mM phenylmethylsulfonyl fluoride (PMSF)]. After freezing and thawing, cells were disrupted by sonication on ice. The cell lysate was centrifuged at 32,000×*g* at 4 °C for 30 min after solubilization by 1% Triton-X. The supernatant was applied to Chelating Sepharose Fast Flow (GE Healthcare) and washed with extraction buffer containing 20, 40, and 60 mM imidazole. The bound protein was eluted with elution buffer [10 mM Tris-HCl (pH 7.5) containing 5% glycerol, 100 mM NaCl, 500 mM imidazole, 1 mM DTT, and 1 mM PMSF], followed by dialysis with storage buffer [50 mM Tris-HCl (pH 7.4) containing 100 mM NaCl, 14 mM mercaptoethanol, and 10% glycerol]. Purified proteins were stored at − 80 °C before use.

### Gel mobility shift assay for the DNA binding of KsgA

The substrate oligonucleotides used in the gel mobility shift assay are shown in Fig. 3(a). Oligonucleotides were labeled with [γ-^32^P] ATP at the 5′-end by T4 polynucleotide kinase (TOYOBO, Japan) and then annealed to complementary oligonucleotides in buffer containing 10 mM HEPES-KOH pH 7.5 and 50 mM NaCl. The gel mobility shift assay was performed at 4 °C in a reaction mixture (10 μl) containing 25 mM Tris-HCl (pH 8.0), 500 μM DTT, 500 μM ethylenediaminetetraacetic acid (EDTA), 10% glycerol, 25 mM NaCl, 25 mM KCl, 10 μM ZnCl_2_, 125 μM of each dNTP, 4 mM spermidine, 500 ng calf thymus DNA, 10 fmol of the ^32^P-labeled double-stranded oligonucleotide, and 100 pmol of purified protein. After the reaction, samples were loaded onto 12% polyacrylamide gels in 90 mM Tris-borate (pH 8.3) containing 50 mM EDTA. After electrophoresis at 100 V, gels were exposed to imaging plates and analyzed on a BAS-1800 Image Analyzer (Fuji Film).

### Construction of antibody

For preparation of recombinant GST-tagged KsgA, *E. coli* BL21 (DE3) were introduced with the pGEX-4 T-3-*ksgA* plasmid vector. GST-KsgA protein expression was induced by the addition of 0.1 mM IPTG. GST-KsgA was purified with a glutathione-sepharose 4B column (GE Healthcare) and then the GST-tag was removed with thrombin. Antiserum was prepared by immunizing rabbits with the purified KsgA protein (Keari Inc., Japan). Affinity purification was carried out by binding to the purified KsgA protein blotted onto nitrocellulose membrane. Buffer was substituted to PBS supplemented with 0.1% BSA, 0.1% sodium azide and 50% glycerol.

### Immunoblotting

The *E. coli* CC101 *mutM mutY ksgA* (*mutM*::Km *mutY*::Tet *ksgA*::Km) [27] strain was introduced with pYP73 plasmids with genes coding KsgA or KsgA (1-213). CC101 *mutM mutY ksgA* with these plasmids was grown overnight at 37 °C to stationary phase in LB medium containing 0.1 mM IPTG and the appropriate antibiotics. Cells were harvested and resuspended in lysis buffer [10 mM Tris-HCl (pH 7.5) containing 5% glycerol, 100 mM NaCl, 1 mM dithiothreitol (DTT), and 1 mM PMSF]. After freezing and thawing, cells were disrupted by sonication on ice to obtain crude extracts. The protein concentrations of crude extracts were measured using the BCA Protein Assay Kit (Pierce). 2x sample buffer [4% SDS, 100 mM Tris-HCl (pH 6.8), 20% glycerol, 12% beta-mercaptoethanol, and a small amount of BPB] was added to crude extracts and purified proteins and they were subjected to 12% SDS-PAGE. Proteins were transferred to a nitrocellulose membrane and stained by amido black. Then, the membrane was blocked with 5% non-fat milk in PBST (PBS/0.05% Tween 20) for 20 min at room temperature. The membrane was incubated overnight at 4 °C with anti-KsgA antibody (purification in this study, 1:1), and then reacted with rabbit-IgG-HRP (sc-2030, Santa Cruz Biotechnology, 1:5000) for 1 h at room temperature. The membrane was washed with PBST after incubation with each antibody. Bound antibodies were visualized by chemiluminescence (ECL; Amersham or SuperSignal West Pico Chemiluminescent; Thermo fisher scientific). The membrane was exposed to X-ray film (FUJIFILM). Images of X-ray film were analyzed by ImageJ 1.53 (Wayne Rasband National Institute of Health, USA http://imagej.nih.gov/ij).

### Measurement of spontaneous mutant frequency

The *E. coli* CC101 *mutM mutY ksgA* (*mutM*::Km *mutY*::Tet *ksgA*::Km) [27] strain was introduced with pYP73 plasmids with or without genes coding KsgA or KsgA (1-213). CC101 *mutM mutY ksgA* with these plasmids was grown overnight at 37 °C to stationary phase in LB medium containing 0.1 mM IPTG and the appropriate antibiotics. We spread 100 μl of the overnight culture, 10-times diluted culture or 10^2^-times diluted culture on LB plate containing 100 μg/ml rifampicin (Nacalai Tesque) respectively. After incubating the plates at 37 °C for 24 h, there were several to hundreds of colonies per rifampicin plate. Then, we calculated the average number per undiluted, which was used as the number of the rifampicin-resistant cells. To assess viable cells, 10^6^-times diluted overnight culture or 10^7^-times diluted that were plated on LB plate each, followed by an incubation at 37 °C for 24 h. There were tens to hundreds colonies per LB plate. We calculated the average number per undiluted, which was used as the number of the viable cells. The spontaneous mutant frequency was determined by the following equation.

$$\textrm{spontaneous}\ \textrm{mutant}\ \textrm{frequency}=\frac{\textrm{the}\ \textrm{number}\ \textrm{of}\ \textrm{the}\ \textrm{rifampicin}-\textrm{resistant}\ \textrm{cells}}{\textrm{the}\ \textrm{number}\ \textrm{of}\ \textrm{the}\ \textrm{viable}\ \textrm{cells}}\times {10}^8$$

### Kasugamycin sensitivity assay

*E. coli* AB1157 *ksgA* (*ksgA*::Km) was introduced with pYP73 plasmids with or without genes coding KsgA or KsgA (1-213). AB1157 and AB1157 *ksgA* with these plasmids were grown overnight at 37 °C in LB media with 100 μg/ml ampicillin (Nacalai Tesque). Overnight cultures of these bacterial transformants were diluted 100-fold in fresh LB media and allowed to grow to an OD_600_ of 0.2 at 37 °C. Cultures were diluted 20-times in fresh LB media. One hundred microliters of 0.1 mM IPTG and 800 μg/ml kasugamycin (Wako Pure Chemical Industries, Ltd.) were added to the cultures. Cultures were grown at 37 °C and OD_600_ was measured after 15 h.

### Statistical analysis

Data for statistical analyses were from more than three independent experiments. Data are presented as the mean ± standard error of the mean (s.e.m.). The significance of differences between conditions was analyzed by a one-way ANOVA with the Tukey-Kramer test using R 3.5.1. See https://www.r-project.org/ for more information about this version. *P* values < 0.05 were considered to be significant.

## Results

### The C terminus of KsgA has a similar structure to H2TH domains of MutM and Nei

**Fig. 1:**
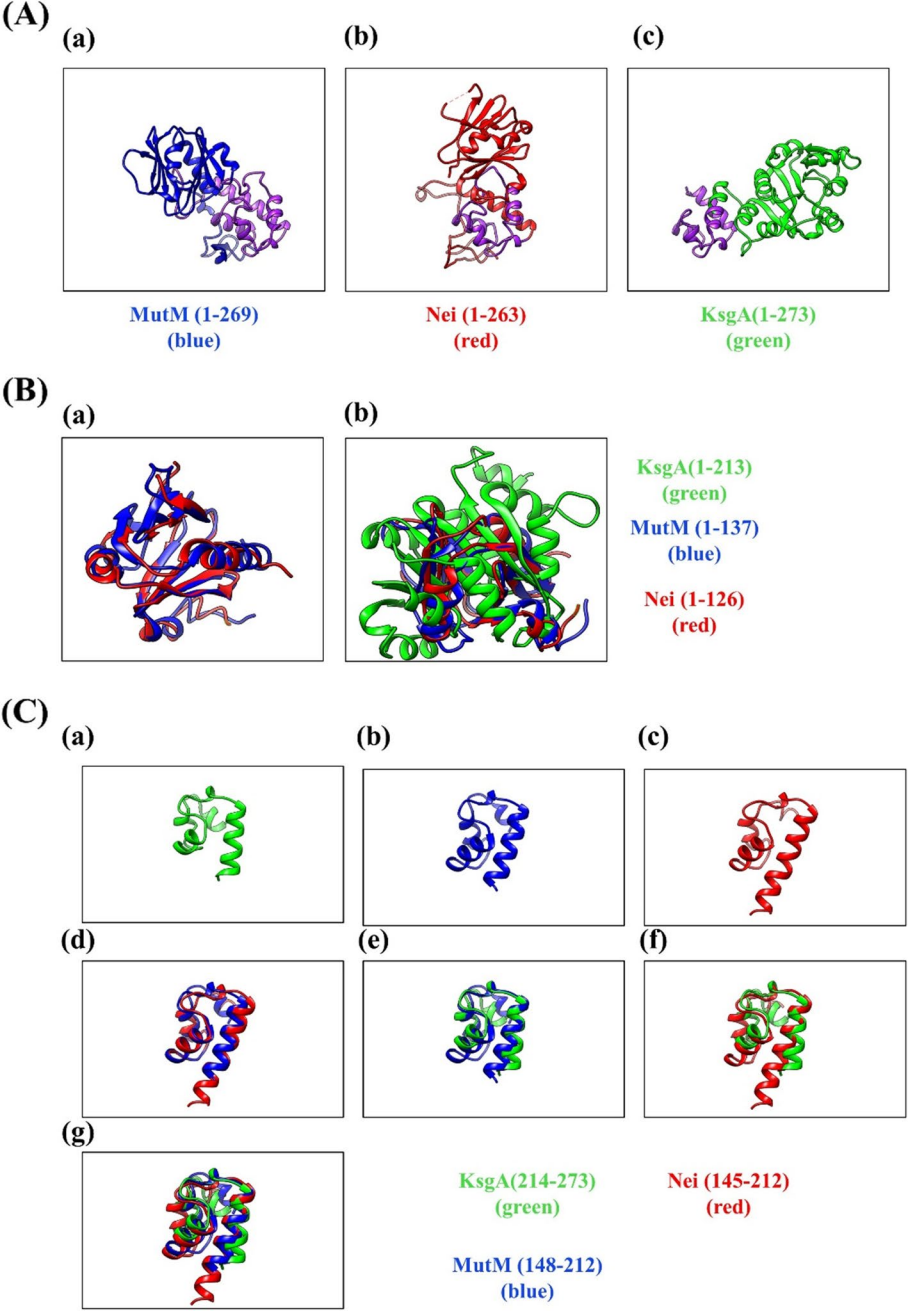
Comparisons of structures and sequences of KsgA, MutM, and Nei. Green = KsgA (PDBID: 1QYR); Red = Nei (PDBID: 1Q39); Blue = MutM (PDBID: 1R2Z). (**A**) The 3D structures of (**a**) MutM, (**b**) Nei, and (**c**) KsgA. Purple indicates the (**a**) H2TH domain of MutM, (**b**) H2TH domain of Nei, and (**c**) amino acids 214-273 of KsgA. (**B**) Comparison N-termini of proteins. (**a**) Comparison of MutM (1-137) and Nei (1-126). (**b**) Comparison of MutM (1-137), Nei (1-126), and KsgA (1-213). (**C**) Comparison of C-termini of proteins. (**a**) KsgA (214-273); (**b**) MutM (148-212); (**c**) Nei (145-212); (**d**) superimposition of MutM (148-212) and Nei (145-212); (**e**) superimposition of KsgA (214-273) and MutM (148-212); (**f**) superimposition of KsgA (214-273) and Nei (145-212); (**g**) superimposition of KsgA (214-273), MutM (148-212), and Nei (145-212)

Residues 129-229 of MutM (full length 269 amino acids) and 126-189 of Nei (full length 263 amino acids) constitute the H2TH domain that binds to DNA in order to repair damaged bases [15, 43, 44]. A previous study reported that residues 214-273 of KsgA (full length 273 amino acids) were sequentially homologous to amino acids 148-212 of MutM and amino acids 145-212 of Nei [27]. However, it currently remains unclear whether the 3D structure of the C terminus of KsgA is similar to that of the H2TH domains of MutM and Nei. To investigate similarities in the C terminus of KsgA to the H2TH domains of MutM and Nei, we compared their 3D structures. We obtained 3D structural data on MutM, Nei, and KsgA from PDBj, and visualized them using UCSF Chimera (Fig. 1(A)). The 3D structures of the C termini of MutM (148-212), Nei (145-212), and KsgA (214-273) were then compared (Fig. 1(C)). We used the root mean square deviation (RMSD) as the scale to measure structural similarities. As shown in Table 1, the pruned atom pairs of the C termini of KsgA and MutM and that of the C termini of KsgA and Nei were 25 (across all 54 pairs) and 21 (across all 55 pairs), respectively. In comparisons of the N-termini of MutM, Nei, and KsgA (Fig. 1(B)), the pruned atom pairs of the N termini of MutM (1-137) and Nei (1-126) was 55 (across all 119 pairs), suggesting they have similar structures. On the other hand, UCSF Chimera was unable to find atom pairs in comparisons of the N terminus of MutM with that of KsgA (Fewer than 3 residues). Additionally, UCSF Chimera could find only 5 pruned atom pairs (across all 92 pairs) in comparisons of the N terminus of Nei with that of KsgA. This showed that the C terminus of KsgA has a structure similar to H2TH domains of MutM and Nei while the N terminus of KsgA does not.

**Table 1:** Comparison of similarity between each protein

**Protein name 1**	**MutM(148-212)**	**MutM (148-212)**	**Nei(145-212)**	**MutM(1-137)**	**MutM(1-137)**	**Nei(1-126)**
**Protein name 2**	Nei (145-212)	KsgA (214-273)	KsgA (214-273)	Nei (1-126)	KsgA (1-213)	KsgA (1-213)
**# of pruned atom pairs** **(RMSD)**	44 (0.927 Å)	25 (1.067 Å)	21 (1.188 Å)	55 (1.119 Å)	–	5 (1.385 Å)
**# of all pairs** **(RMSD)**	59 (2.493 Å)	54 (6.644 Å)	55 (7.477 Å)	119 (3.088 Å)	Fewer than 3	92 (15.770 Å)
**Percentage of pruned atom pairs in all pairs**	75%	46%	38%	46%	–	5%

These data were calculated by UCSF Chimera. All pairs are all the atom pairs that UCSF Chimera was able to compare between two proteins. Pruned atom pairs are the atom pairs which the value of RMSD was less than 2 Å among all pairs

### Expression and purification of full-length KsgA and C-terminal-deficient and N-terminal-deficient recombinant proteins

**Fig. 2:**
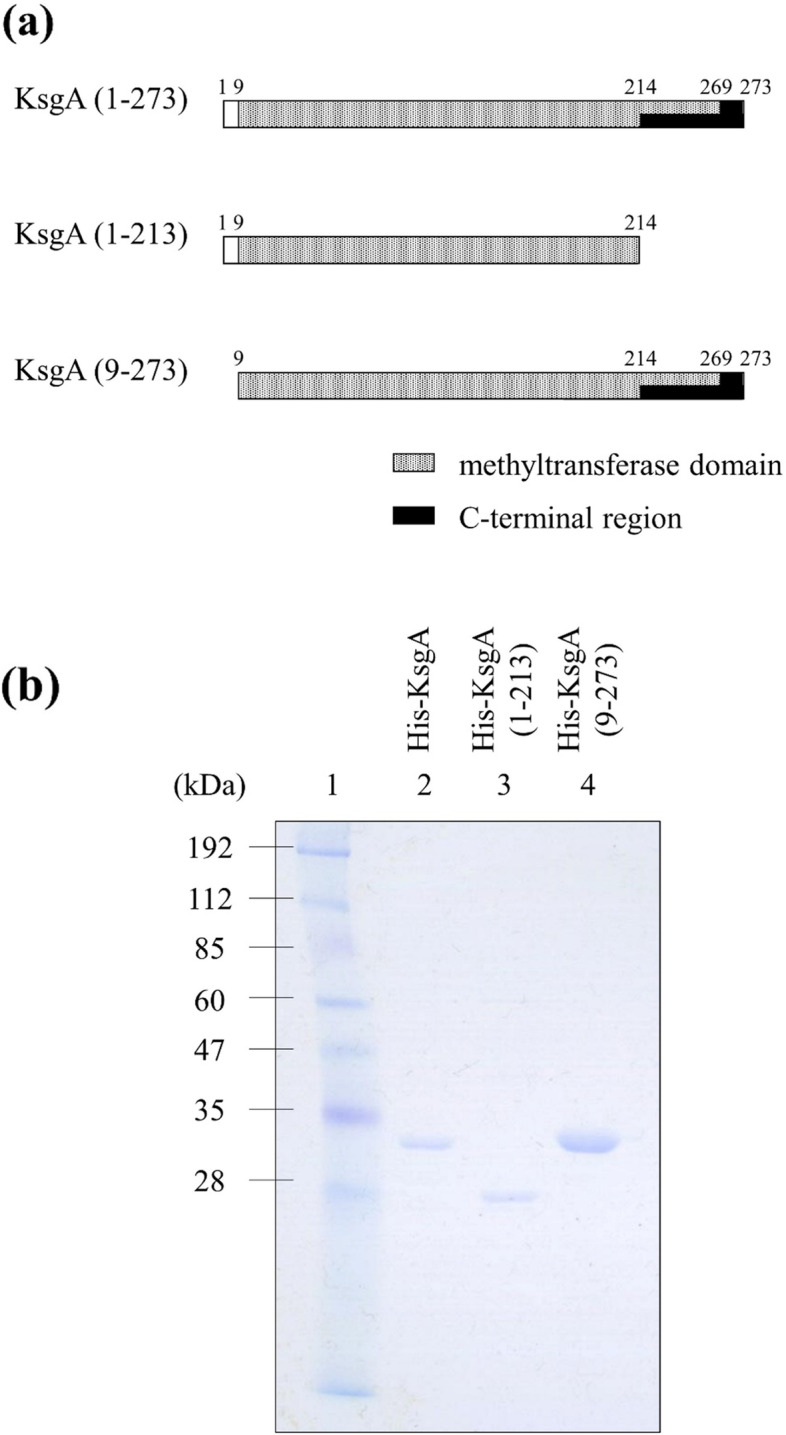
Three types of recombinant KsgA and their purification. (**a**) Domain organization of full-length KsgA, KsgA (1-213), and KsgA (9-273). (**b**) Purification of *E. coli* full-length KsgA, KsgA (1-213), and KsgA (9-273). *Escherichia coli* BL21(DE3) carrying pET21a (+)-KsgA, pET21a (+)-KsgA (1-213), or pET21a (+)-KsgA (9-273) was induced by 0.1 mM IPTG at 20 °C for 16 h. Proteins were separated by 0.1% SDS-12% PAGE and stained with Coomassie Blue. Lane 1, molecular weight marker; lane 2, purified His-KsgA (19.5 μg); lane 3, purified His-KsgA (1-213) (7.3 μg); lane 4, His-KsgA (9-273) (85.3 μg)

Since the 214-273 amino acid residues of KsgA were suggested to be structurally homologous to the H2TH domain, we generated KsgA lacking this C terminus region and examined its activity. The gene coding KsgA lacking residues 214-273 was amplified by PCR and the fragment was subcloned into the plasmid vector pET21a (+) to obtain His-tagged fusion proteins. Using the same method, the genes coding full-length KsgA and KsgA lacking amino acid residues 1-8 in the N terminus were amplified by PCR and the fragments were subcloned into the same vector. Therefore, three types of His-tagged proteins: full-length KsgA, one without amino acids 214-273 in the C terminus of KsgA, and one without amino acids 1-8 in the N terminus (Fig. 2(a)), were used in subsequent experiments. Fusion proteins were expressed in *E. coli* BL21(DE3), and pET21a (+)-KsgA, pET21a (+)-KsgA (1-213), or pET21a (+)-KsgA (9-273) was induced by IPTG and purified by Chelating Sepharose Fast Flow (GE Healthcare). The 31-kDa His-KsgA protein, 25-kDa His-KsgA (1-213) protein, and 30-kDa His-KsgA (9-273) protein were detected on a 0.1% SDS-12% polyacrylamide gel (Fig. 2(b)).

### C-terminal-deficient KsgA lost its DNA-binding activity

**Fig. 3:**
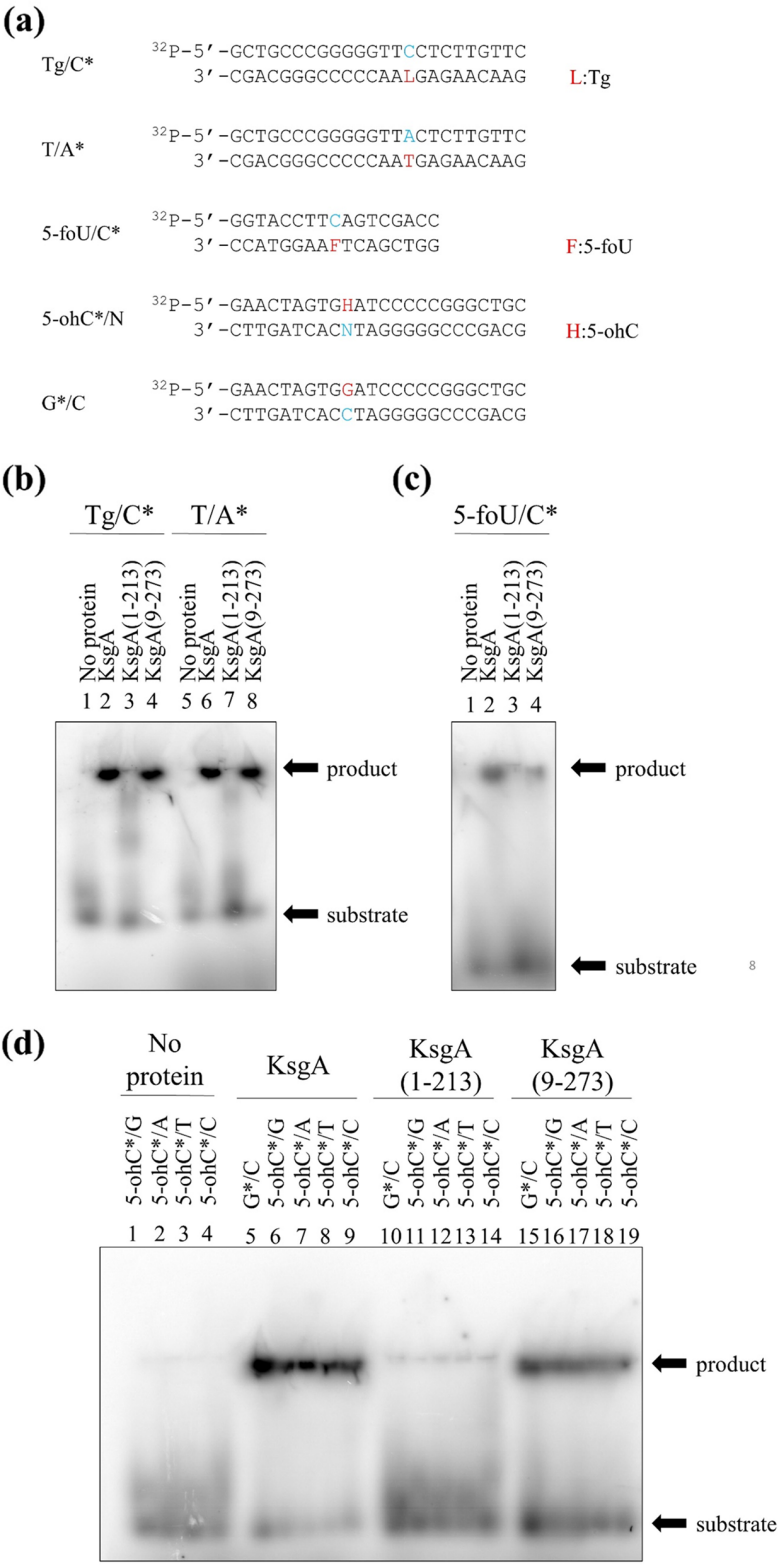
Complex of purified KsgA, KsgA (1-213), or KsgA (9-273) with double-stranded oligonucleotides. Oligonucleotide substrates (10 fmol) were incubated at 4 °C for 60 min with purified proteins (100 pmol). Samples were separated by 12% PAGE. (**a**) Oligonucleotides used in the gel mobility shift assay. The asterisk indicates the base is present on the DNA strand labelled with ^32^P. The red letter means a damaged base or its replacement with a normal base, and the blue letter means a base that is a pair of the red letter base. Oligonucleotides contain (**b**) Tg/C (lanes 1-4) and T/A (lanes 5-8), (**c**) 5-foU/C (lanes 1-4), and (**d**) 5-ohC/G (lanes 1, 6, 11, and 16), 5-ohC/A (lanes 2, 7, 12, and 17), 5-ohC/T (lanes 3, 8, 13, and 18), 5-ohC/C (lanes 4, 9, 14, and 19), and G/C (lanes 5, 10, and 15). (**b**) Lanes 1 and 5, no protein; lanes 2 and 6, purified His-KsgA; lanes 3 and 7, purified His-KsgA (1-213); lanes 4 and 8, purified His-KsgA (9-273). (**c**) Lane 1, no protein; lane 2, purified His-KsgA; lane 3, purified His-KsgA (1-213); lane 4, purified His-KsgA (9-273). (**d**) Lanes 1-4, no protein; lanes 5-9; purified His-KsgA; lanes 10-14, purified His-KsgA (1-213); lanes 15-19, purified His-KsgA (9-273)

To clarify whether KsgA proteins bind to DNA, the gel mobility shift assay was performed. Oligonucleotides containing 5-foU/C or Tg/C were selected because KsgA was previously reported to exhibit DNA glycosylase activity [27]. Oligonucleotides with the same sequence, except the damaged base that was replaced with a normal base, were also prepared. Figure 3(a) shows the oligonucleotides used in this assay. ^32^P-labeled double-stranded oligonucleotides were incubated at 4 °C for 60 min with purified His-KsgA (1-273), His-KsgA (1-213), or His-KsgA (9-273). The results obtained showed that KsgA (1-273) and KsgA (9-273) bound to oligonucleotides containing Tg/C (Fig. 3(b), lanes 2 and 4) and 5-foU/C (Fig. 3(c), lanes 2 and 4). However, the complex of KsgA (1-213) and oligonucleotides was not detected (Fig. 3(b), lane 3 and Fig. 3(c), lane 3). In addition, oligonucleotides containing 5-ohC were investigated because the Nei protein recognizes and repairs 5-ohC [45]. As shown in Fig. 3(d), His-KsgA (1-273) and His-KsgA (9-273) bound to oligonucleotides containing 5-ohC paired with G, A, T, or C (lanes 6-9 and 15-19, respectively), whereas KsgA (1-213) did not (lanes 10-14).

### ksgA lacking the C-terminal region did not suppress the spontaneous mutant frequency of the *E. coli* mutM mutY ksgA mutant

**Fig. 4:**
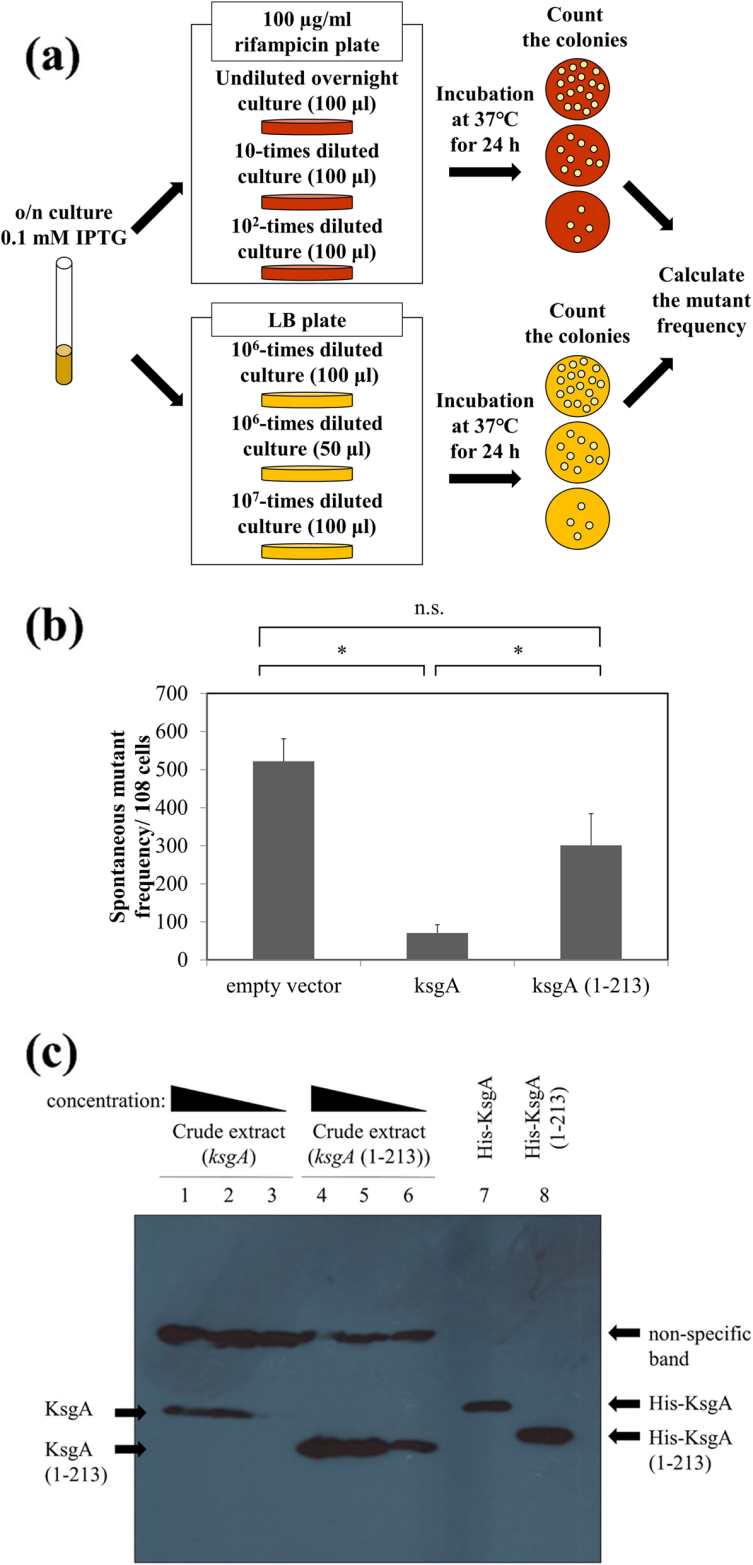
Frequency of spontaneous rifampicin mutation in DNA glycosylase-deficient (CC101 *mutM mutY ksgA*) *E. coli*. Cells were introduced with the empty vector (pYP73), *ksgA* gene, or *ksgA* (1-213) gene. (**a**) Procedure for measuring spontaneous mutation frequencies. Each strain of *E.coli* was grown overnight at 37 °C in LB medium containing 0.1 mM IPTG and the appropriate antibiotics. The undiluted or diluted culture were plated on LB plate or that containing rifampicin and incubated 37 °C for 24 h. We counted the colonies and calculated the number of the viable cells and the rifampicin-resistant cells and determined the spontaneous mutant frequency from these numbers. (**b**) The result of spontaneous mutant frequency measurement. Mean + S.E. values of six independent experiments are shown (*n* = 6). An asterisk (*) indicates *p* values < 0.05. (**c**) Comparison of expression levels by western blotting. Crude extracts were obtained from *E.coli* and quantified by BCA method. Crude extracts and purified proteins were separated by 12% SDS-PAGE and transferred to nitrocellulose membrane. Lanes 1-3, crude extract from *E.coli* introduced with *ksgA* gene (88, 44, and 18 μg, respectively); lanes 4-6, that with *ksgA* (1-213) gene (88, 44, and 18 μg, respectively); lane 7, purified His-KsgA (0.75 μg); lane 8, purified His-KsgA (1-213) (0.75 μg)

A previous study reported that KsgA exhibited DNA glycosylase activity and a deficiency in the *ksgA* gene increased the spontaneous mutant frequency [27]. To investigate the effects of the C-terminus deletion of KsgA, we measured the spontaneous mutant frequency of the *E. coli mutM mutY ksgA* strain introduced with pYP73 (empty vector), pYP73-*ksgA*, or pYP73-*ksgA* (1-213) by counting and calculating rifampicin-resistant mutants. The reason we used *mutM* strains is that MutM has DNA glycosylase activity to remove 5-foU [22]. Since KsgA removes cytosine opposite to 5-foU, defects in both *mutM* and *ksgA* causes more spontaneous mutation than defect in *ksgA* alone. Additionally, according to our previous studies, *mutY* deficiency increases spontaneous mutant frequency by about 8 ~ 10 fold in both *mutM ksgA* and *ksgA* [27]. Since this assay is subject to large variations, we decided to use *mutY* deletion in order to minimize the effect of the variation. The spontaneous mutant frequency of the mutant with the empty vector was 521 (± 59) per 10^8^ cells, whereas that of bacteria transformed with full-length *ksgA* was 71 (± 21) per 10^8^ cells, which was significantly lower. On the other hand, the mutant frequency in *E. coli mutM mutY ksgA* transformed with *ksgA* (1-213) was 301 (± 83) per 10^8^ cells, which was not significantly different from that in *mutM mutY ksgA* with the empty vector. Additionally, we examined the expression levels of full-length or C terminus-deficient *ksgA* gene in *E.coli* introduced with pYP73-*ksgA* or pYP73-*ksgA* (1-213) by western blotting (Fig. 4(c)). The same amounts of proteins were applied to the gel for electrophoresis after quantifying by the BCA method and we confirmed that the similar amounts of proteins were transferred to the membrane using amido black staining. The expression level of *ksgA* (1-213) is not less than that of *ksgA*, as shown in Fig. 4(c). Quantification of the bands by ImageJ showed that KsgA (1-213) was more than 2 times expressed compared with KsgA.

### ksgA lacking the C-terminal region rescued dimethyltransferase activity in ksgA-deficient cells

**Fig. 5:**
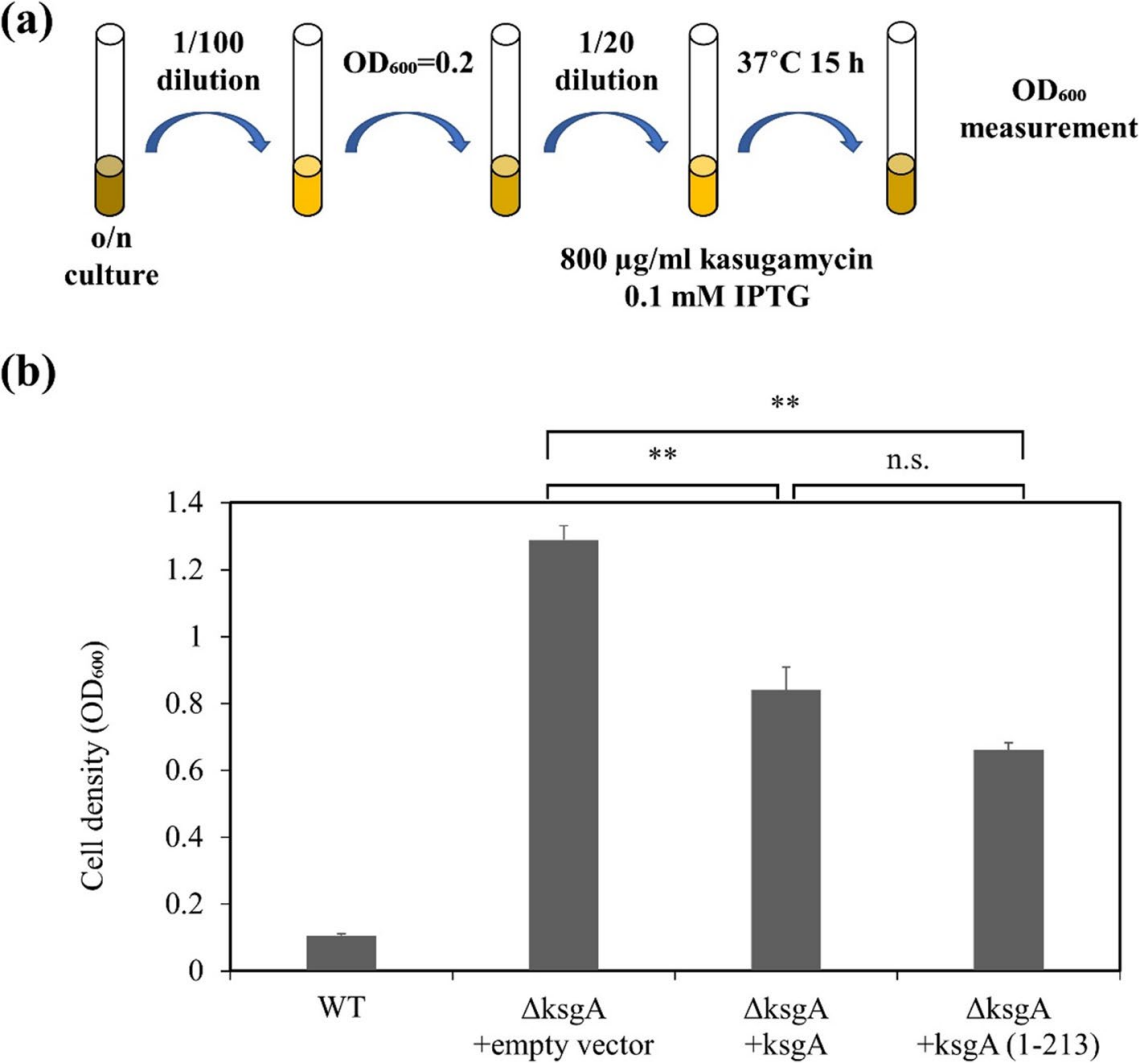
Functional complementation of KsgA rRNA methyltransferase activity by KsgA (1-213) in WT (AB1157) and *ksgA*-deficient (AB1157 *ksgA*) *E. coli* strains. (**a**) Procedure of measuring kasugamycin-resistance. Each strain of *E.coli* was grown overnight at 37 °C in LB medium containing antibiotics. 10^2^-times diluted overnight culture was incubated at 37 °C to an OD_600_ of 0.2 and then diluted 20-times in LB medium containing IPTG and kasugamycin. After incubation at 37 °C for 15 h, the growth of cells was estimated by measuring OD_600_. (**b**) The result of kasugamycin assay. Mean + S.E. values of three independent experiments are shown (*n* = 3). Two asterisks (**) indicates *p* values < 0.01

The kasugamycin assay was performed to assess the dimethyltransferase activity of KsgA (1-213). In *E. coli*, 16S rRNA matures through dimethylation by KsgA. Kasugamycin interferes with the survival of *E. coli* by inhibiting the function of 16S rRNA. However, in the *ksgA*-deficient strain, rRNA is not modified and the immature 16S rRNA is produced, which is not inhibited by kasugamycin [32]. *E. coli* AB1157 *ksgA* was transformed with the empty vector or plasmid carrying *ksgA* or *ksgA* (1-213). As shown in Fig. 5, the OD_600_ of AB1157 and AB1157 *ksgA* with the empty vector were approximately 0.104 and 1.289, respectively. This is consistent with previous findings showing that the *ksgA*-deficient strain was more resistant to kasugamycin than the wild-type strain [30, 31]. In AB1157 *ksgA* with the ksgA gene, OD_600_ was approximately 0.841, suggesting that the defect in *ksgA* was rescued by the transformation of pYP73-*ksgA*. The OD_600_ of AB1157 *ksgA* with pYP73-*ksgA* (1-213) was approximately 0.662. This was significantly different from that of AB1157 *ksgA* with the empty vector.

## Discussion

KsgA was originally identified in *E. coli* as a dimethyltransferase that regulates translation by modifying rRNA [30–34]. KsgA is also a bifunctional DNA glycosylase that exhibits DNA glycosylase and AP lyase activities that target the opposite sides of oxidized thymine [27]. A previous study revealed that the C-terminal sequence of KsgA was similar to the H2TH domains of MutM and Nei [27]. However, it currently remains unclear whether the C terminus of KsgA functions in its DNA-binding activity. Therefore, we investigated the structure of the C terminus of KsgA and performed a gel mobility shift assay and complementation assay using C-terminal-deficient KsgA.

We acquired 3D structure data on KsgA, MutM, and Nei (Fig. 1(A)) and compared their structures using UCSF Chimera. Structural comparisons revealed that the percentage of pruned atom pairs in all pairs of the C termini of KsgA and MutM and those of KsgA and Nei were 46 and 38%, suggesting similarities not only in sequences, but also in structures (Fig. 1(C)). On the other hand, UCSF Chimera could not find atom pairs in comparisons of the N terminus of MutM with that of KsgA. Also, only 5 pruned atom pairs across all 92 pairs was found in comparisons of the N termini of KsgA and Nei (Table 1), suggesting that KsgA has no structure in its N terminus that is common with that of MutM or Nei.

In contrast to KsgA, MutM and Nei have a common N-terminal region (Fig. 1(B)). Members of the Fpg/Nei superfamily have N-terminal proline or valine residues, which are involved in nucleophilic attacks at the C1′ position of the target nucleotide [46–48]. Most DNA glycosylases in the Fpg/Nei superfamily also have a zinc finger motif in the C terminus to bind to DNA [44]. KsgA does not have these common sequences (Fig. 1(B)), which suggests that it is a new type of DNA glycosylase. In this case, the H2TH domain, which was considered to be unique to the Fpg/Nei superfamily, may be shared among other families and the importance of this domain will increase.

In the gel mobility shift assay to assess the DNA-binding activity of the C terminus of KsgA, we purified full-length KsgA and KsgA without C-terminal amino acids 214-273. Amino acids residues 1-8 of KsgA were not the part of the methyltransferase domain (Fig. 2(a)) (https://www.genome.jp/tools/motif/) and N-terminal amino acids were identified as the active center of DNA glycosylase activity in MutM and Nei [16–18]. Therefore, we also purified KsgA without N-terminal amino acids 1-8 in the N terminus.

The gel mobility shift assay demonstrated that full-length KsgA and N-terminal-deficient KsgA (9-273) bound to duplex oligonucleotides containing damaged bases (Fig. 3). On the other hand, KsgA (1-213) lacking the C terminus failed to bind to DNA, suggesting that amino acids 214-273 of the C terminus are involved in DNA binding (Fig. 3). The N termini of MutM and Nei are the active center and not involved in DNA binding. Similarly, the N terminus of KsgA may not be required for DNA binding. His-KsgA and His-KsgA (9-273) also bound to undamaged oligonucleotides (Fig. 3(b), lanes 6 and 8 and Fig. 3(d), lanes 5 and 15). This is consistent with the findings of a previous study that examined the DNA-binding activity of KsgA [49]. Lee and Wallace reported that the DNA glycosylases MutM and Nei bound to undamaged DNA and moved along the DNA backbone to seek damaged DNA [50]. KsgA may employ the same approach to identify its target bases.

To investigate the role of the C terminus of KsgA in vivo, we performed rescue experiments using the rifampicin resistance mutant assay system and *ksgA*-deficient strains. The results of the mutation assay showed that full-length KsgA suppressed spontaneous mutations in the *rpoB* gene, whereas KsgA lacking amino acids 214-273 did not (Fig. 4(b)). The reason why KsgA (1-213) failed to suppress spontaneous mutations in *E. coli mutM mutY ksgA* is not due to low expression levels (Fig. 4(c)). These results indicate that the C terminus of KsgA is important for the suppression of spontaneous mutations.

We also examined the effects of the loss of the C-terminal domain on dimethyltransferase activity. Full-length KsgA and KsgA (1-213) both reduced the high kasugamycin resistance of *ksgA*-deficient strains (Fig. 5). These results indicate that amino acids 214-273 were unrelated to dimethyltransferase activity (Fig. 5). Partially restoration of kasugamycin resistance by *ksgA* or *ksgA* (1-213) might be due to the fact that the expression of the gene in the plasmid is not consistent with the native expression in cells.

In addition, the lack of spontaneous mutation suppression by *ksgA* (1-213) in Fig. 4(b) was not due to loss of dimethyltransferase activity.

Other ribosomal proteins have DNA repair functions. S3 is a component of the 40S ribosomal subunit, and has been shown to exhibit AP endonuclease activity and positively regulate nucleotide excision repair in association with the TFIIH complex [41, 51]. The 60S acidic ribosomal protein P0 is another ribosomal protein that has been reported to interact with APEX1 and exhibit AP endonuclease activity [40]. Similarly, KsgA is a ribosomal protein that exhibits the activities of two enzymes, DNA glycosylase and dimethyltransferase.

Therefore, since several ribosomal proteins have been reported to exhibit enzymatic activity related to BER, an interaction between protein translation and BER is expected to exist that warrants further study. We herein demonstrated that the C terminus of KsgA was necessary for DNA-binding activity, not dimethyltransferase activity. The identification of the site involved in BER in the ribosomal protein KsgA will lead to a more detailed understanding of the mechanisms by which similar ribosomal proteins are involved in BER. We hope that this tool will provide insights into the mechanisms by which ribosomal proteins are involved in DNA repair.

## Conclusion

The present study revealed that the C-terminal amino acids of KsgA are highly similar to those of the H2TH structural domain, which is essential for DNA-binding function and the inhibition of spontaneous mutations.

## Data Availability

All data generated or analyzed during the present study are included in this published article.

## Abbreviations

AP site: Apurinic/apyrimidinic site
BER: Base excision repair
DTT: Dithiothreitol
EDTA: Ethylenediaminetetraacetic acid
H2TH domain: Helix-2turn-helix domain
IPTG: Isopropyl-D-1- thiogalactopyranoside
LB: Luria-Bertani
PMSF: Phenylmethylsulfonyl fluoride
OD_600_: Optical density at 600 nm
RMSD: Root mean square deviation
UCSF: University of California, San Francisco
